# Bridging Mechanism and Therapy in Ocular Disease: Mitochondrial, Vascular, and Immune Pathways in the Eye

**DOI:** 10.3390/life16101624

**Published:** 2026-09-28

**Authors:** Sheryene Tejeda, Sese-Owei Ekaye, Jean M. Taylor, Maya Sharma

**Affiliations:** Medicinal Technologies, Philadelphia, PA 19102, USA; seseowei.ekaye@uniben.edu (S.-O.E.); support@medicinaltechnologies.com (J.M.T.); mayasharma@medicinaltechnologies.com (M.S.)

**Keywords:** Fuchs endothelial corneal dystrophy, diabetic macular edema, age-related macular degeneration, optic neuritis, mitochondrial dysfunction, complement system, TCF4 trinucleotide repeat, optical coherence tomography angiography, artificial intelligence

## Abstract

There is an increasing global burden of ocular disorders, with an estimated 43.3 million people worldwide currently blind and a further 295 million living with moderate-to-severe vision impairment, driven by a complex pathogenesis that involves multiple cascade failures ranging from mitochondrial dysfunction to chronic neuroinflammation and blood–retinal barrier breakdown. This narrative review synthesizes peer-reviewed literature identified through targeted searches of PubMed/MEDLINE, Embase, and the Cochrane Library, prioritizing publications from the last five years and supplemented by foundational studies where necessary, to examine molecular mechanisms underlying both anterior and posterior segment pathologies, including Fuchs endothelial corneal dystrophy (FECD), diabetic macular edema (DME), age-related macular degeneration (AMD), and antibody-mediated optic neuritis. We link the most recent clinical insights into the structural abnormalities of these disorders with translational research advances, examining how genetic drivers such as the TCF4 trinucleotide repeat expansion, vascular endothelial growth factor (VEGF) signaling, complement system dysregulation, and antibody-specific neuroinflammatory injury converge on shared cellular vulnerabilities across otherwise distinct ocular tissues, while explicitly noting where this convergence is well evidenced and where it remains hypothesis-generating. We also evaluate how novel imaging methods, including optical coherence tomography (OCT) and OCT angiography (OCTA), together with emerging artificial intelligence (AI)-assisted diagnostic tools, are reshaping early disease detection. Within these four index conditions, we examine therapeutic classes across a spectrum of evidentiary maturity, from complement inhibitors and anti-VEGF agents already altering clinical management of AMD and DME, to gene therapy and mitochondrial-stabilizing strategies that remain at the preclinical or early-investigational stage; adjacent pharmacological strategies whose supporting evidence was generated outside these four conditions are addressed separately in an appendix rather than treated as part of the core evidence base. To make the degree of mechanistic convergence explicit rather than assumed, we constructed a four-domain evidentiary matrix grading mitochondrial, microvascular/barrier, complement/innate immune, and neuroinflammatory evidence for each condition; no single condition showed strong evidence across all four domains, and the four conditions differed meaningfully in which domains dominated their pathogenesis. As a narrative rather than systematic review with a non-systematic search strategy and single-reviewer evidence grading, this synthesis should be read with those limitations in mind. Decoding complex networks of cellular and systemic processes in ocular disease remains critical to developing high-efficiency therapeutics that could potentially preserve ocular cellular homeostasis and halt progressive visual decline.

## 1. Introduction

Vision loss and vision impairment disorders represent critical socio-economic challenges and burdens for modern healthcare. Aging populations are affected disproportionately due to the rising life expectancy on the planet. The pathophysiologies underlying visual decline vary among patient groups; candidate shared mechanisms proposed across this heterogeneous group include oxidative stress, chronic inflammation, metabolic disturbance, and microvascular dysfunction, though, as this review will show, no single condition draws strong support across all of these candidate domains, and the degree of overlap differs meaningfully by condition (see Table 1). Sustaining ocular homeostasis requires the regulation of numerous biological functions that range from the active pump activity of anterior corneal endothelium to metabolic activity of the multilayered posterior retina.

The eye is an anatomically compact and, at the same time, highly heterogeneous organ comprising not only avascular innervated tissues of the anterior segment but also an extremely metabolically active and highly vascularized posterior segment. Such heterogeneity implies that disease mechanisms should be considered individually for each part of the eye, taking into account the specific vulnerabilities of the tissues. For example, the inability of corneal endothelial cells to regenerate and the high energetic demands of the neuroretina should both be considered when relating ocular pathological changes to the whole organism, which includes systemic factors such as glucose control, vasoactivity, and whole-body inflammatory activity.

Historically, clinical management relied on broad-spectrum anti-inflammatory drugs or surgery, including endothelial keratoplasty and repeated intravitreal injections. Although this approach has proven successful at slowing disease progression, it is usually applied only after considerable structural damage has already occurred. New opportunities are now arising: the use of high-resolution imaging techniques together with more thorough molecular characterization is shifting the paradigm toward early-stage cellular protection and metabolic preservation, allowing intervention before irreparable damage is done. This review discusses the key mechanisms of ocular diseases along with their biomarkers and promising therapeutic approaches that may halt degenerative processes before serious visual impairment occurs.

The following diseases were chosen for analysis: Fuchs endothelial corneal dystrophy, diabetic macular edema, age-related macular degeneration, and antibody-mediated optic neuritis, because they involve different parts of the eye (both anterior and posterior) while demonstrating distinctive but related mechanisms of disease development, including mitochondrial dysfunction, breakdown of the blood–retinal barrier, extracellular matrix instability, and immune-mediated neurodegeneration. Other major ocular diseases, including glaucoma, inherited retinal degeneration, and dry eye disease, share overlapping oxidative and mitochondrial biology with the conditions discussed here and could plausibly be analyzed within the same comparative framework; they were excluded solely to keep the scope of this review tractable, a decision made independently of any commercial interest of the sponsoring organization.

### Epidemiological and Public Health Context

The scale of vision loss provides essential context for why mechanistic research in ophthalmology carries such weight. According to the most recent Global Burden of Disease (GBD) analysis available at the whole-population level, an estimated 43.3 million people worldwide are blind and a further 295 million people live with moderate-to-severe vision impairment; both figures are projected to rise substantially by 2050 as the global population ages [18,19]. These overall blindness and vision-impairment figures derive from the GBD 2019 data cycle, whereas the AMD-specific figures presented below derive from the more recent GBD 2021 cycle; the two are not directly comparable year-for-year, and this discrepancy reflects the GBD study’s staggered disease-specific update schedule rather than an inconsistency in the underlying data [18,19,20]. Blindness and vision impairment already impose an estimated US$411 billion in annual global productivity losses, a burden that falls disproportionately on lower- and middle-income regions where access to specialized ophthalmic care remains limited; this estimate is based on 2018 economic and demographic data combined with 2020 vision-impairment prevalence data, expressed in 2018 US dollars at purchasing power parity [21,22].

Age-related macular degeneration alone illustrates how quickly this burden can shift. Between 1990 and 2021, the global number of people with vision impairment attributable to AMD more than doubled, rising from approximately 3.6 million to 8.1 million, and disability-adjusted life-years attributable to the disease increased by 91% over the same period [20]. Projections indicate that vision impairment attributable to AMD specifically (as distinct from overall AMD prevalence, which is substantially higher) could reach approximately 21.3 million people by 2050 absent meaningful changes in risk-factor exposure, although modeling suggests that reducing tobacco use as a modifiable risk factor could meaningfully offset this trajectory [20]. Diabetic eye disease follows a broadly similar pattern: as global diabetes prevalence continues to climb, the population at risk for diabetic macular edema grows in parallel, compounding demand on already overburdened retinal-care systems, a theme explored further below.

These trends matter for two reasons beyond their public-health weight. First, they underscore that the four conditions examined in this review are not rare curiosities but represent some of the most common drivers of preventable or treatable vision loss encountered in everyday ophthalmic practice. Second, they clarify why the field has increasingly prioritized early, mechanism-based intervention over reactive, damage-stage treatment: a modest delay in disease progression, multiplied across a population numbering in the hundreds of millions, represents a meaningful reduction in global disability. The remainder of this review’s emphasis on convergent pathogenic mechanisms and their associated biomarkers is intended to support exactly that shift, by clarifying where in the disease course intervention is likely to be both mechanistically justified and practically feasible.

## 2. Materials and Methods

This review provides a narrative synthesis of the cellular and molecular mechanisms underlying four ocular and neuro-ophthalmic conditions affecting the anterior and posterior segments of the eye: Fuchs endothelial corneal dystrophy (FECD), diabetic macular edema (DME), age-related macular degeneration (AMD), and antibody-mediated optic neuritis (AQP4-ON/MOG-ON). SANRA (the Scale for the Assessment of Narrative Review Articles) [23] is a quality appraisal instrument rather than a reporting guideline; it is used here as a drafting checklist rather than a claimed reporting standard, and this review’s structure was designed with reference to the quality domains it assesses-justification of the review’s importance and aims, description of the literature search, referencing of statements, presentation of the evidence base for key claims, and appropriate scientific reasoning. SANRA does not require, and this review does not claim, adherence to PRISMA-level systematic-review methodology.

Literature was identified through targeted searches of three biomedical databases: PubMed/MEDLINE, Embase, and the Cochrane Library, consistent with the databases named in the Abstract. Searches were first conducted in November 2025 and were re-run and extended in August 2026 to capture literature published during the revision period; no lower date limit was applied to the databases themselves, but retrieved records were screened with precedence given to publications from the preceding five years (2021–2026), supplemented by earlier foundational or landmark studies (e.g., original genetic-association or trial reports) where necessary for mechanistic or historical context. Searches were restricted to English-language publications in peer-reviewed journals; conference abstracts, preprints, and non-peer-reviewed sources were excluded except where explicitly identified as such (e.g., company press releases cited only for clinical-trial status updates, which are distinguished from peer-reviewed evidence throughout the text).

Search terms were organized into topic clusters corresponding to the four index conditions and the cross-cutting themes addressed in this update (epidemiology, AI-assisted diagnostics, and complement- and gene-based therapeutics). Representative Boolean search strings for each cluster, together with the specific database interfaces used, are provided in Table S1 for reproducibility; not every cluster was searched across all three databases; the imaging/AI-diagnostics cluster was searched in PubMed/MEDLINE and Embase only, and the epidemiology/global-burden cluster in PubMed/MEDLINE only, since the Cochrane Library’s focus on intervention trials made it a low-yield source for those two topic areas specifically, while all four disease-mechanism clusters and the complement/gene-therapy cluster were searched across all three databases as stated in the Abstract. Records were included if they reported original human clinical, human tissue/cell-model, or high-relevance preclinical data, or systematic/narrative review synthesis, on the cellular, molecular, structural, or therapeutic aspects of one or more of the four index conditions; records were excluded if they addressed an ocular condition outside the four index conditions without direct mechanistic relevance to a domain examined here, if they were available only as an abstract without a retrievable full text, or if they duplicated data already captured in a more recent or more directly relevant source. Across the three databases, the authors’ best reconstruction is that searches returned on the order of 600–650 unique records after de-duplication, of which roughly 100–120 underwent full-text review after title/abstract screening for mechanistic or translational relevance; 58 sources were retained in the final reference list, which is an exact count rather than a reconstruction. The upstream screening figures are a post hoc reconstruction of the search process, not a contemporaneously logged count, and consistent with the non-systematic nature of this review disclosed in the Discussion, should be read as order-of-magnitude approximations rather than a formally audited PRISMA-style accounting.

Findings were grouped by shared pathological mechanism: mitochondrial and metabolic failure, microvascular and barrier impairment, complement and innate immune activation, and neuroinflammation and extracellular matrix (ECM) alterations. Because this update also addresses epidemiological context, artificial-intelligence-assisted diagnostics, and complement-targeted and gene-based therapeutics, supplementary searches specifically covered these areas (see Table S1 for the corresponding search strings), alongside the disease- and mechanism-specific terms listed above.

## 3. Pathogenic Mechanisms in Anterior and Posterior Segment Conditions

### 3.1. Comparative Mechanistic Framework

Before examining each condition individually, we define the comparative framework used throughout this review. Rather than asserting convergence in general terms, we specify four mechanistic domains a priori—mitochondrial/metabolic dysfunction, microvascular/barrier compromise, complement/innate immune activation, and neuroinflammatory injury—and evaluate the strength of evidence for each domain’s involvement in each of the four conditions under review. We define convergence operationally as concordant molecular pathway activation demonstrated in the relevant human cell type or tissue, not merely the use of shared vocabulary such as “oxidative stress” or “inflammation.” Evidence for each domain-condition pairing is graded as Strong (direct evidence in the relevant human cell type or tissue, including clinical trial data where applicable), Moderate (mechanistic or indirect human evidence), Preliminary (limited, indirect, or largely preclinical evidence), or Not identified in this review (no substantive evidence identified in the non-systematic literature search underlying this review). Table 1 summarizes this assessment.

We use the label “Not identified in this review” rather than “Not established” advisedly: because the search underlying this review is explicitly non-systematic (Section 2), the absence of a domain from our sample cannot be distinguished from the true absence of evidence in the broader literature. The relabeled tier is intended to make this scope limitation explicit at the point where a reader encounters it, rather than only in the Discussion.

This matrix makes explicit what the narrative sections below only imply: none of the four conditions shows Strong evidence across all four domains, and the conditions differ meaningfully in which domains dominate their pathogenesis. FECD shows Strong evidence in two linked domains (mitochondrial dysfunction and the endothelial barrier failure it produces), with no substantive evidence identified in the remaining two. Antibody-mediated optic neuritis shows Strong evidence in a single domain (neuroinflammatory injury), Preliminary evidence in two others (complement activation and, from mouse-model data only, microvascular compromise), and no substantive evidence in the fourth. DME and AMD each show Strong evidence in one domain (microvascular/barrier compromise and complement/innate immune activation, respectively) alongside Moderate or Preliminary evidence in others, making them the more genuinely multi-domain conditions of the four. Framed this way, the convergence claim that motivates this review is best understood as convergence on a shared set of candidate mechanistic domains, not as evidence that all four conditions share the same dominant pathway. Glaucoma, inherited retinal degeneration, and dry eye disease, briefly noted in the Section 1 as sharing overlapping biology with the conditions reviewed here, were not scored in this matrix because a full literature assessment of their evidence base was outside the scope of this review. Figure 1 presents this same evidentiary assessment schematically, mapping each of the four mechanistic domains onto each of the four conditions with line weight indicating evidence strength.

### 3.2. Mitochondrial Dysfunction and Metabolic Failure

As one of the organ structures with the highest energy demands, the eye is highly vulnerable to mitochondrial dysfunction. The corneal endothelium, for example, performs its critical function of corneal deturgescence (the relative dehydration necessary for good vision) through active ion transport via Na+/K+-ATPase pumps. Because human corneal endothelial cells have little capacity for proliferation, their energy demands must be met over a lifetime without meaningful cellular replacement.

In Fuchs endothelial corneal dystrophy (FECD), degeneration of the corneal endothelial cells is associated with increased mitochondrial stress. Energetic failure leads to an oxidant-antioxidant imbalance that damages mitochondrial DNA and triggers a persistent unfolded protein response (UPR) [1]. When mitophagy fails to clear damaged organelles, cellular structure becomes compromised, leading to corneal edema and vision impairment [1].

This vulnerability to mitochondrial dysfunction in the cornea is not unique; it reflects a broader rule of ocular physiology—any tissue with high energy demands and little regenerative potential is vulnerable to oxidative injury. The retinal pigment epithelium and photoreceptors of the posterior segment are further examples, since constant mitochondrial function is required for phototransduction and outer-segment renewal.

The trigger for this mitochondrial vulnerability in FECD is, in a majority of cases, now understood to be genetic. Approximately 70% of FECD cases in the United States and other predominantly European-ancestry populations are attributable to an intronic CTG trinucleotide repeat expansion (CTG18.1) within the TCF4 gene; this attributable fraction is substantially lower in East Asian cohorts, at approximately 44% in a Singapore Chinese population and even lower in Japanese cohorts, indicating that non-CTG18.1 mechanisms contribute more heavily to FECD outside European-ancestry populations [2,24]. In the United States and other predominantly European-ancestry populations, the disease remains the leading indication for corneal transplantation, though population-specific registry data would be needed to confirm this ranking globally [2]. Several non-mutually exclusive mechanisms have been proposed to link this repeat expansion to endothelial cell loss: dysregulation of TCF4 expression itself, toxic gain-of-function effects from repeat-containing RNA that accumulates as nuclear foci within the corneal endothelium, non-canonical translation of the repeat sequence into toxic peptide products, and progressive somatic instability of the repeat length with age [2]. Each of these mechanisms converges on the same downstream vulnerability described above: mitochondrial stress and failure of protein quality control in a cell population that cannot be meaningfully replaced once lost.

Ong Tone and colleagues have described this convergence as a self-perpetuating ‘vicious cycle’: oxidative and endoplasmic reticulum stress triggered by the repeat expansion impair mitochondrial bioenergetics, which in turn increases oxidative burden and further destabilizes the unfolded protein response, accelerating endothelial cell loss over years to decades [3]. This framing carries practical relevance for therapeutic development, since it suggests that interventions capable of interrupting the cycle at any single point—for example, by reducing oxidative stress, supporting mitochondrial quality control, or directly targeting the repeat-containing RNA transcript—could plausibly slow disease progression regardless of exactly where in the cycle they act. It also helps explain the characteristically slow, decades-long clinical course of FECD, because the pathological process is cyclical and self-amplifying rather than the product of a single acute insult, symptoms often do not become apparent until a substantial fraction of the endothelial cell population has already been irreversibly lost, a pattern of delayed clinical detection that recurs throughout the conditions discussed in this review.

### 3.3. Microvascular Integrity and Systemic Fluid Regulation

In the posterior segment, vision depends on the integrity of the retinal microvasculature and the functionality of the blood–retinal barrier (BRB). In diabetic macular edema, chronic metabolic damage disrupts endothelial tight junctions and contributes to fluid accumulation across the macular layers [25]. This pattern reflects a broader feature of diabetic microvascular disease, which produces vascular changes driven by chronic hyperglycemia across multiple organs, including the kidney and peripheral nerves. DME can therefore be viewed as a local manifestation of a systemic vascular disorder.

Recent clinical observations suggest that DME may not be a purely local ophthalmic phenomenon. A single cross-sectional study of 100 eyes from 100 patients with type 2 diabetes found that phase angle (PhA), a bioimpedance-derived measure of cell membrane integrity, was lower in patients with diabetic retinopathy and DME (5.45 ± 0.84 degrees) than in those without retinopathy (6.69 ± 0.69 degrees, *p* < 0.001) or with retinopathy but no DME (6.05 ± 1.15 degrees, *p* = 0.032), and that eyes with a higher burden of hyper-reflective foci had a significantly higher extracellular-to-total-body-water ratio (*p* = 0.001) [25]. These findings are associative rather than mechanistic: the study cannot establish whether systemic fluid status contributes causally to retinal fluid accumulation, and the study’s own authors noted that the between-group PhA differences, while statistically significant, were small in absolute terms and had not been shown to be clinically meaningful. The finding is preliminary and awaits replication in larger, longitudinal cohorts before it should inform clinical practice or be treated as an established systemic contributor to DME.

The central molecular driver of fluid accumulation in DME is vascular endothelial growth factor (VEGF), which is upregulated in the diabetic retina in response to chronic hyperglycemia, tissue hypoxia, and the accumulation of advanced glycation end-products [6]. VEGF signaling directly destabilizes the tight-junction proteins that seal the inner blood–retinal barrier, increasing paracellular permeability and driving fluid into the neurosensory retina [6,7]. This mechanistic insight underlies the current standard of care: intravitreal anti-VEGF therapy is now first-line treatment for vision-threatening DME and has transformed outcomes for a disease that previously carried a much higher risk of irreversible central vision loss, a conclusion established directly by the pivotal RISE and RIDE phase 3 randomized trials of ranibizumab, in which 34–46% of treated eyes across the two trials’ 0.3 mg and 0.5 mg dosing arms gained at least 15 letters of visual acuity at 24 months compared with 12–18% of sham-treated eyes [7,8,26]. Head-to-head comparative-effectiveness data exist among the three original anti-VEGF agents: the DRCR.net Protocol T trial found that at 1 year, aflibercept produced greater mean visual acuity gains than bevacizumab or ranibizumab (+13.3 vs. +9.7 vs. +11.2 letters, respectively), though the trial investigators themselves noted this overall difference was driven entirely by eyes with worse baseline visual acuity and was not clinically meaningful across the full study population; by 2 years, aflibercept’s advantage over ranibizumab was no longer apparent, though its advantage over bevacizumab persisted in the worse-baseline-vision subgroup [27]. More recently, faricimab, a bispecific antibody that inhibits both VEGF-A and angiopoietin-2, demonstrated non-inferior visual acuity gains and better anatomical outcomes (greater reduction in central subfield thickness) compared with aflibercept dosed every 8 weeks in the YOSEMITE and RHINE phase 3 trials, while allowing roughly three-quarters of patients to extend to 12-week or longer dosing intervals via a personalized treatment-interval regimen, representing the first dual-mechanism approach to reach approval for DME [28]. Intravitreal corticosteroids, which act on inflammatory rather than purely angiogenic pathways, remain an important second-line option, particularly for eyes with a suboptimal anti-VEGF response, although their use is tempered by a higher risk of cataract progression and intraocular pressure elevation [26].

Sustaining the benefit of anti-VEGF therapy over time has proven to be a distinct clinical challenge from achieving it initially. Because DME is a chronic disease requiring repeated intravitreal injections over years, treatment burden and adherence have emerged as significant barriers to maintaining the vision gains achieved early in therapy. Treat-to-target strategies that titrate injection frequency to macular morphology on OCT, rather than applying a fixed dosing interval, have been proposed as one way to balance efficacy against the cumulative burden of frequent clinic visits [29].

Several gene-therapy programs designed to provide sustained endogenous anti-VEGF expression following a single administration are now in clinical development for DME. Registration details for each program below were independently verified against ClinicalTrials.gov. ADVM-022 (ixoberogene soroparvovec), an AAV.7m8-vectored aflibercept construct, was evaluated in the Phase 2 INFINITY trial (ClinicalTrials.gov NCT04418427) for DME; the trial was unmasked early after a Suspected Unexpected Serious Adverse Reaction of hypotony with panuveitis and vision loss was reported in a high-dose recipient, and additional high-dose patients subsequently developed steroid-refractory intraocular pressure decreases [29,30]. The sponsor has since discontinued further development of ADVM-022 specifically for DME, while continuing development of the same molecule for neovascular AMD, where the safety profile has differed [31]. 4D-150, a dual-transgene intravitreal AAV vector expressing aflibercept and a VEGF-C-targeted microRNA, is registered for DME in the ongoing Phase 2 SPECTRA trial (NCT05930561); 60-week interim results reported in 2025 were characterized by the sponsor as positive, and the sponsor has since reported alignment with the European Medicines Agency on a registrational pathway for 4D-150 in DME [32]. RGX-314, delivered subretinally, is registered for DME in the Phase 2 ELAAVATE trial (NCT06942520); this registration was verified against the ClinicalTrials.gov record, which confirms the condition studied as center-involved diabetic macular edema (CI-DME), not neovascular AMD-RGX-314 is separately being evaluated for neovascular AMD in other, differently named trials (e.g., ATMOSPHERE, ASCENT, AAVIATE), which are outside the scope of the DME discussion here [33]. A fourth RGX-314 program, ALTITUDE (ClinicalTrials.gov NCT04567550), delivers the same molecule via in-office suprachoroidal injection rather than subretinal delivery, and is evaluating diabetic retinopathy and DME as a less invasive alternative administration route to ELAAVATE’s subretinal approach; both are part of the sponsor’s broader RGX-314 development program for diabetic eye disease [33]. Taken together, these four programs illustrate a genuine divergence in outcome to date: one program’s DME development has been discontinued following a distinct and serious safety signal, while the other three remain in active development, underscoring that a treatment-focused review of this therapeutic class must weigh program-specific safety data rather than treating gene-therapy delivery of anti-VEGF as a single undifferentiated risk category.

At the cellular level, recent work has connected the vascular story in DME back to the mitochondrial vulnerability discussed earlier in this review. High-glucose exposure impairs mitophagy—the selective autophagic clearance of damaged mitochondria—in the cell types that constitute the inner blood–retinal barrier, and this impairment is temporally and mechanistically associated with tight-junction breakdown, though the primary experimental evidence for strict upstream causality is stronger for mitochondrial fission driving endothelial injury generally than for tight-junction loss specifically. This distinction matters at the level of the specific proteins studied: Fis1 and Drp1 are core components of the mitochondrial fission machinery, a process that is upstream of, and mechanistically linked to, mitophagy but is not itself mitophagy—the two should not be treated as interchangeable. In rat retinal endothelial cells cultured in high-glucose medium (30 mM, versus 5 mM normal-glucose controls), siRNA-mediated knockdown of Fis1 and Drp1 reduced high-glucose-induced apoptosis and preserved mitochondrial respiratory function, directly demonstrating that manipulating mitochondrial fission causally alters downstream high-glucose injury in this cell type, independent of any change in glucose level itself [4,5]. This experiment demonstrates an effect on mitochondrial fission and apoptosis specifically; it does not directly measure mitophagic flux, and it should not be read as direct evidence that mitophagy itself, rather than fission, is causally upstream of tight-junction loss. Whether the broader mitophagy-failure hypothesis holds specifically for tight-junction protein loss, as opposed to fission-associated apoptosis, has not been established with equivalent causal (as opposed to correlative) evidence in the literature reviewed here. Restoring effective mitophagy has therefore been proposed as a therapeutic target distinct from, and potentially complementary to, VEGF blockade, since it addresses barrier stability at its structural origin rather than only its most visible angiogenic consequence [4]. This connection illustrates a broader theme of this review: the mitochondrial dysfunction that drives corneal endothelial loss in FECD and the microvascular barrier failure that drives DME both implicate impaired mitochondrial quality control as a contributing vulnerability, expressed differently depending on the metabolic demands and regenerative capacity of the tissue involved. This parallel should not be overstated: the corneal endothelium in FECD is a non-regenerating, post-mitotic cell population in which mitochondrial failure directly and progressively depletes an irreplaceable cell pool, whereas the retinal microvascular endothelium in DME is a distinct, non-post-mitotic cell type in a continuously perfused vascular bed, in which the same category of mitochondrial quality control failure contributes to junctional and functional injury without the identical irreplaceability constraint. The two conditions are therefore better described as sharing a category of cellular vulnerability-impaired mitochondrial quality control-than as manifestations of a single shared mechanism in a single shared cell type.

Diabetic microvascular disease is also, as noted above, rarely confined to the eye. The same chronic hyperglycemic and inflammatory processes that damage the retinal microvasculature act on the vasculature of the kidney, peripheral nerves, and other end organs, producing a shared pattern of pericyte loss, basement membrane thickening, and progressive microvascular occlusion [7]. This broader systemic vulnerability is well established for diabetic microvascular disease generally, independent of the preliminary bioimpedance findings discussed above.

### 3.4. Neurodegenerative Pathways and Neuroinflammation

Functioning signal transduction pathways and ECM integrity are similarly relevant to retinal health. Signaling proteins such as Reelin participate in retinal development, waste processing, and maintenance of synapses within the retinal pigment epithelium (RPE) and photoreceptors [34].

Whether disruption of these signaling pathways directly contributes to the development of AMD remains an open mechanistic question rather than an established finding. What is established is narrower: amyloid-beta-containing aggregates accumulate within a subset of lipid-filled drusen deposits [35]. This is a narrower and better-supported claim than the broader assertion that drusen contain misfolded proteins generally: amyloid-beta assemblies are a documented, characterized component of drusen in AMD-affected eyes, but this does not establish that protein misfolding is a general feature of drusen composition, nor that Reelin-pathway disruption specifically drives this process. This chronic neuroinflammatory pattern parallels degenerative processes observed in Alzheimer’s disease, underscoring the eye’s potential value as a window onto neurodegeneration more broadly.

The parallels between AMD and central nervous system (CNS) neurodegeneration extend beyond protein-aggregation biology. Degeneration in both the CNS and the retina involves chronic, low-level activation of resident immune cells, microglia in the CNS and their retinal equivalents. These cells normally serve a protective function by clearing harmful material, but continue to damage tissue if they remain persistently active. This shared immunology has motivated the use of retinal disorders as models for developing therapies aimed at other neurodegenerative diseases, and vice versa.

Beyond the Reelin-associated signaling changes described above, the most extensively characterized driver of AMD pathogenesis is dysregulation of the complement system, a component of innate immunity ordinarily responsible for opsonizing pathogens and clearing cellular debris. Genome-wide association studies identified variants in complement factor H (CFH) and complement factor I (CFI) as major genetic risk factors for AMD more than a decade ago—a landmark 2005 genome-wide screen found that homozygosity for the CFH risk allele increased AMD likelihood by a factor of 7.4—and subsequent immunohistochemical work confirmed that complement proteins C3, C5, and the membrane attack complex accumulate directly within drusen deposits [9,36]. Under normal conditions, a set of regulatory proteins keeps complement activation localized and self-limiting; in AMD, this regulation appears to fail in a tissue-specific way, allowing chronic, low-grade complement activation to persist in the macula even though systemic complement activity remains largely normal [9,10].

This mechanistic understanding motivated a wave of complement-targeted drug development, most notably against the terminal complement components C3 and C5, which sit at a convergence point for both the classical and alternative complement pathways [10]. Pegcetacoplan, a pegylated cyclic peptide that binds and blocks the cleavage of C3, was the first complement inhibitor to demonstrate a measurable reduction in the growth rate of geographic atrophy lesions in randomized controlled trials—the phase 3 OAKS and DERBY trials reported reductions in lesion growth of roughly 20% with monthly dosing over 24 months—although its effect on functional visual outcomes has been more modest than its effect on anatomical lesion growth [11,12,37,38]. A 2023 Cochrane review pooling these trials rated the certainty of evidence for any meaningful benefit on functional or quality-of-life outcomes at one year as little to none, and separately rated pegcetacoplan’s association with increased macular neovascularization as low-certainty evidence and its association with increased endophthalmitis risk as moderate-certainty evidence [38]. Avacincaptad pegol, a C5 inhibitor, demonstrated a comparable reduction in lesion growth in the phase 3 GATHER2 trial [13]-though this comparison should be read cautiously, since GATHER2’s headline result is a 12-month readout, whereas the pegcetacoplan figure above reflects 24-month follow-up, and lesion-growth reduction is cumulative over time rather than constant, so the two percentages are not directly equivalent without further normalization, which the same Cochrane review rated as moderate-certainty evidence, noting that the effect estimate may still change with further research [38]. Regulatory status in Europe has followed a similar path for both agents: like pegcetacoplan, avacincaptad pegol’s EU marketing authorization application was withdrawn by its sponsor in October 2024 following unfavorable interactions with the EMA’s Committee for Medicinal Products for Human Use, so neither complement inhibitor is currently approved in the European Union, despite both being FDA-approved in the United States [39]. This gap between anatomical and functional benefit has prompted a broader reconsideration of AMD pathogenesis: if complement inhibition alone is not sufficient to fully arrest vision loss, other converging mechanisms are likely also contributing meaningfully to disease progression [40].

Two such mechanisms have received particular recent attention. The first is dysregulated lipid metabolism: the retina is unusually lipid-rich, and oxidized lipid deposits together with altered high-density lipoprotein profiles appear to both promote and be promoted by local complement activation, creating a self-reinforcing cycle analogous to the mitochondrial–oxidative stress cycle described earlier in FECD. The second is immunosenescence and ‘inflammaging’—the age-related decline in immune system regulation that shifts the retina and choroid from a homeostatic, low-level ‘para-inflammatory’ state into a chronic, unresolved inflammatory state involving both innate and adaptive immune cell populations [40]. Because immunosenescence is a feature of aging generally rather than a mechanism unique to the eye, this framing further reinforces the parallel drawn earlier between AMD and neurodegenerative diseases of the central nervous system, both of which appear to share an underlying vulnerability to age-related loss of immune homeostasis.

Taken together, these findings suggest that AMD therapeutics targeting a single node—whether complement, lipid metabolism, or angiogenesis—are likely to offer only partial benefit, and that combination approaches addressing multiple convergent pathways simultaneously may ultimately be required to meaningfully alter the disease’s long-term trajectory [11,40].

## 4. Advanced Biomarkers and Diagnostic Modalities

Early disease detection requires objective, structural biomarkers capable of identifying pathology before symptomatic vision loss occurs. Optical coherence tomography (OCT) and its angiographic counterpart (OCTA) have substantially advanced diagnostic capability in ophthalmology, allowing clinicians to visualize microstructural retinal and choroidal changes non-invasively and in real time, at a resolution previously achievable only through histological examination.

Building on the mechanistic domains defined in Table 1, high-resolution, non-invasive OCT and OCTA imaging allow the structural consequences of these domains-corneal edema in FECD, macular edema and drusen formation in DME and AMD-to be tracked over time and used to guide targeted metabolic [41], botanical [42], and neuroprotective [34] interventions.

In neuro-ophthalmic conditions such as optic neuritis, distinguishing between underlying etiologies is clinically essential. Multiple sclerosis-associated optic neuritis (MS-ON) is mediated primarily by T-cell-driven demyelination and is mechanistically distinct from aquaporin-4 antibody-mediated optic neuritis (AQP4-ON) and myelin oligodendrocyte glycoprotein antibody-associated disease (MOGAD), which are genuinely antibody-mediated; grouping MS-ON under an antibody-mediated framework, as earlier optic neuritis nomenclature sometimes did, is no longer consistent with current consensus classification [43,44]. The corticosteroid treatment paradigm for acute MS-ON itself rests on primary randomized-trial evidence: the Optic Neuritis Treatment Trial established that intravenous methylprednisolone, but not oral prednisone alone, speeds visual recovery without altering six-month visual outcomes; the same trial also found that oral prednisone alone, at the dose and duration studied, increased the risk of a new attack of optic neuritis in either eye relative to placebo (relative risk 1.79, 95% CI 1.08–2.95), a finding that led to oral prednisone monotherapy at this dose being considered contraindicated for acute optic neuritis [45]. Differentiating MS-ON, AQP4-ON, and MOG-antibody-associated optic neuritis (MOG-ON) directly informs treatment selection, since each subtype carries a distinct risk of relapse, a distinct expected pattern of visual recovery, and a distinct long-term immunosuppressive treatment strategy. Quantitative OCT reveals distinct diagnostic signatures, including rapid and severe ganglion cell layer loss in AQP4-ON compared with the more variable structural preservation observed in other subtypes [15].

This distinction has become considerably more granular with the accumulation of comparative OCT data across the three major optic neuritis subtypes. In a retrospective multicenter cohort, peripapillary retinal nerve fiber layer (RNFL) thickness measured during the acute attack (before antibody serology typically returns) differentiated MOG-antibody-associated disease (MOGAD) from multiple sclerosis-associated optic neuritis with a receiver-operating-characteristic area under the curve of 0.81: MOGAD attacks showed a median pRNFL increase of 45.5 µm, with 87% of eyes increasing by at least 10 µm, compared with a median increase of only 7 µm in MS-associated attacks, of which roughly one-third exceeded 10 µm [16]. This acute thickening—reflecting optic disk edema—occurs more frequently and more severely in MOGAD than in AQP4-positive neuromyelitis optica spectrum disorder (NMOSD) or MS-associated optic neuritis [16]. The source study did not report adherence to the OSCAR-IB quality control criteria or APOSTEL reporting guidelines commonly used in more recent OCT research, a limitation worth noting when comparing its effect sizes against studies that do report these standards. Paradoxically, this acute thickening is followed, over the following months, by a pattern of chronic thinning that tends to be less severe in MOGAD than in AQP4-ON, such that MOGAD-associated optic neuritis frequently shows better long-term structural and visual recovery despite a more dramatic acute presentation [16,17]. AQP4-ON, by contrast, is associated with the most severe and least reversible ganglion cell and RNFL loss of the three subtypes, consistent with its more aggressive clinical course and higher relapse risk [17].

These OCT-based distinctions are not merely academic. Because AQP4-ON, MOG-ON, and MS-associated optic neuritis carry meaningfully different relapse risks and require different long-term immunosuppressive strategies, structural imaging that can support rapid subtype differentiation directly informs treatment selection at a stage when antibody serology results may not yet be available [17]. This is an instructive example of a broader principle established throughout this review: structural biomarkers are most clinically valuable not simply when they detect that pathology is present, but when they can distinguish between pathologies that look similar on initial presentation but require substantially different management.

Translational models of optic neuritis (mouse) evaluated with non-invasive OCTA demonstrate that vascular damage occurs early and rapidly during inflammatory episodes, with measurable reductions in capillary vessel density, vessel length, and branch-point density within the intermediate and deep capillary plexuses [14]. These preclinical findings suggest OCTA-derived vascular metrics as candidate early biomarkers of ongoing neuroinflammation and a potential tool for assessing therapeutic effect in future clinical studies—an important consideration, since visual acuity alone may understate ongoing tissue damage—but they have not yet been validated in human optic neuritis, and the translational gap between mouse retinal microvasculature and human disease has not been characterized for this specific biomarker.

### Artificial Intelligence and Automated Image Analysis

The diagnostic value of OCT and OCTA imaging is ultimately limited by the pace at which a human grader can review and interpret it, and this limitation has motivated substantial recent investment in artificial intelligence (AI)-assisted image analysis across ophthalmology. Deep learning models, particularly convolutional neural network architectures, have demonstrated strong performance in classifying OCT scans into the major pathological categories relevant to this review—choroidal neovascularization, drusen, diabetic macular edema, and normal retina—often reaching diagnostic accuracy comparable to trained human graders on curated test sets [46,47]. More recent architectures have extended this capability to multi-class segmentation of retinal fluid and pathology across a wider range of B-scan resolutions and acquisition protocols, addressing an earlier limitation in which models trained on one imaging platform generalized poorly to scans acquired on another [48].

Beyond diagnostic classification, AI models are increasingly being applied to the harder problem of predicting individual treatment response before therapy begins. Machine learning models trained on baseline OCT features combined with systemic metabolic and hematologic markers have shown promising accuracy in predicting which patients with DME are likely to achieve a meaningful anatomical response to anti-VEGF therapy, a capability that could eventually allow clinicians to triage patients toward anti-VEGF, corticosteroid, or combination therapy from the outset rather than by sequential trial and error [49]. This kind of predictive application illustrates how imaging-based AI tools are moving beyond simple pattern recognition and toward genuinely personalized treatment planning, a shift that mirrors the broader move toward mechanism-based, individualized therapy discussed throughout this review [46].

These advances come with important caveats that temper immediate clinical enthusiasm. Model performance frequently degrades when applied to data from imaging devices, patient populations, or disease severities outside the original training distribution, and relatively few AI diagnostic tools in ophthalmology have been prospectively validated in real-world clinical workflows rather than retrospectively on curated datasets [46,50]. The available open-access datasets used to train and benchmark many of these models also remain limited in size and demographic diversity relative to the scale needed for robust generalization, a gap that the field has explicitly identified as a priority for future work [51]. For the diseases discussed in this review, AI-assisted OCT interpretation is therefore best understood at present as a promising complement to, rather than a replacement for, the structural and systemic biomarkers described above; its greatest near-term value likely lies in triaging cases for specialist review and flagging subtle structural changes that might otherwise be missed on routine examination, rather than in fully autonomous diagnosis.

Table 2 consolidates the primary segment, core pathogenic mechanism, key biomarker, and evidence level for each of the four conditions discussed in this review, and reflects the domain-level grading introduced in Table 1.

**Table 2:** Comparative overview of the four ocular conditions discussed in this review. Composite mechanism entries are split into individually graded components (see footnote).

Condition	Primary Ocular Segment	Core Pathogenic Mechanism (Individually Graded)	Key Structural/Systemic Biomarker	Evidence Level (Per Component)	Reference(s)
Fuchs endothelial corneal dystrophy (FECD)	Anterior segment (corneal endothelium)	(a) TCF4 CTG18.1 repeat expansion -> mitochondrial stress/UPR failure: Strong (human tissue histopathology + genetics) (b) Downstream mitophagy failure specifically: Preliminary (mechanistic/preclinical; see Table 3)	Corneal pachymetry; endothelial cell density; guttae on specular/confocal microscopy	(a) Strong; (b) Preliminary	[1,2,3]
Diabetic macular edema (DME)	Posterior segment (macula, retinal microvasculature)	(a) VEGF-driven tight-junction breakdown: Strong (human RCT evidence) (b) Mitochondrial fission/mitophagy failure at the inner blood–retinal barrier: Moderate (mechanistic human-relevant review evidence plus a rat cell model; causal role in tight-junction loss specifically not established, see Section 3.3)	OCT central subfield thickness; hyper-reflective foci	(a) Strong; (b) Moderate	[4,5,6,8,25]
Age-related macular degeneration (AMD)	Posterior segment (RPE, Bruch’s membrane, choroid)	(a) Complement dysregulation: Strong (human genetic + immunohistochemical evidence) (b) Lipid-metabolism abnormalities: Moderate (mechanistic human evidence) (c) Immunosenescence/inflammaging: Moderate (human and model evidence)	Drusen volume on OCT; geographic atrophy lesion area	(a) Strong; (b) Moderate; (c) Moderate	[9,34,40]
Antibody-mediated optic neuritis (AQP4-ON/MOG-ON)	Optic nerve; retinal ganglion cell layer	Antibody-specific immune-mediated axonal and neuronal injury: Strong (human clinical + quantitative OCT evidence)	Peripapillary RNFL thickness; ganglion cell complex; OCTA capillary density	Strong	[15,16,17]

TCF4 = transcription factor 4; RPE = retinal pigment epithelium; AQP4-ON = aquaporin-4 antibody-mediated optic neuritis; MOG-ON = myelin oligodendrocyte glycoprotein antibody-mediated optic neuritis; OCT = optical coherence tomography; OCTA = OCT angiography; RNFL = retinal nerve fiber layer. The Evidence Level tiers used in this table are defined separately from, and should not be conflated with, the domain-condition tiers of Table 1: here, Strong = the specific biomarker-mechanism association shown is supported by human clinical-trial or large human-cohort evidence directly linking the mechanism to the biomarker or outcome; Moderate = supported by mechanistic human tissue/cell-model evidence without RCT-level confirmation; Preliminary = supported only by preclinical, indirect, or small human observational evidence; Not established = no direct evidence identified in the literature reviewed here for this specific association. Composite mechanisms are graded per component rather than as a single entry, so that a Strong evidence component (e.g., VEGF-driven tight-junction breakdown in DME) does not carry a co-listed Preliminary- or Moderate-evidence component (e.g., mitophagy failure) under the same single grade.

**Table 3:** Emerging and mechanism-targeted therapeutic strategies discussed in this review, restricted to strategies with evidence generated within the four index conditions (see Appendix A/Table S2 for adjacent anterior-segment and general ocular strategies). A Harms/Safety column is included below.

Strategy	Primary Molecular Target	Disease Application (Index Conditions Only)	Developmental Stage	Evidence Level	Harms/Safety Considerations	Reference(s)
Complement inhibition (pegcetacoplan, avacincaptad pegol)	Complement C3/C5	AMD (geographic atrophy)	FDA-approved (US); both agents’ EU applications refused/withdrawn (2024)	Strong (multiple Phase 3 human RCTs)	Intraocular inflammation and occlusive retinal vasculitis reported post-marketing (pegcetacoplan); dose-dependent increase in exudative AMD conversion by month 24 (12% monthly, 7% every-other-month, vs. 3% sham)	[10,11,12,13,39]
Sustained/gene-based anti-VEGF delivery (ADVM-022, 4D-150, RGX-314)	VEGF-A	DME	Phase 2 clinical trials (heterogeneous outcomes; see Section 3.3)	Moderate (Phase 2 human trials; one program halted for safety)	ADVM-022: hypotony with panuveitis and vision loss at high dose; DME program discontinued. 4D-150: no comparable safety signal in reported 60-week interim data. RGX-314 (ELAAVATE): trial too early in follow-up for safety conclusions; absence of a reported signal to date should not be read as evidence of a favorable safety profile	[29,30,31,32,33]
Mitophagy/fission-targeted agents	Mitochondrial fission (Fis1/Drp1) and mitophagy quality control machinery	DME; FECD (proposed)	Preclinical	Preliminary (preclinical/mechanistic only)	No human safety data available; preclinical only	[4,5]
AI-assisted treatment response prediction	N/A (diagnostic/decision-support)	DME, AMD	Retrospective validation; early prospective studies	Moderate (retrospective human data; meta-analytic)	Not a direct patient-safety exposure, but misclassification or algorithmic bias could indirectly affect treatment selection; prospective real-world validation still limited	[49]

VEGF = vascular endothelial growth factor; DME = diabetic macular edema; AMD = age-related macular degeneration; FECD = Fuchs endothelial corneal dystrophy; AI = artificial intelligence. Evidence Level here uses a third, strategy-level tier system distinct from both Table 1’s domain-condition tiers and Table 2’s biomarker-mechanism tiers: Strong = supported by multiple Phase 3 human randomized controlled trials; Moderate = supported by Phase 2 human trial data or large retrospective human cohorts; Preliminary = supported only by preclinical, mechanistic, or small early-phase data. This reflects the strength and design of the underlying supporting studies (e.g., Phase 3 RCT vs. preclinical/animal data only) for each therapeutic strategy as a whole, and is distinct from Developmental Stage, which reflects regulatory/clinical-development status. Reference numbers are listed once, in the Reference(s) column only, to avoid duplication with the Evidence Level column.

## 5. Therapeutic Frontiers: Beyond Conventional Approaches

Improving treatment efficiency requires accounting for the specific biochemical, structural, and molecular environment of the ocular region. Rather than simply suppressing inflammation, therapy should target the specific steps in disease development described above: mitochondrial quality control, receptor-mediated signaling, cell metabolism, and inflammation resolution. This section is restricted to therapeutic strategies with evidence generated specifically within the four index conditions. Adjacent anterior-segment and general ocular anti-inflammatory strategies that act on overlapping pathways but have not been evaluated specifically within the four index conditions—endogenous opioid receptor modulation, polyamine-based metabolic support, and botanical/ethnopharmacological anti-inflammatories—are discussed separately in Appendix A.

### 5.1. Rho-Kinase Inhibition in the Anterior Segment

Pharmacological support of corneal endothelial wound healing offers a complementary strategy to the mitochondrial-targeted approaches discussed above for FECD. Topical Rho-associated kinase (ROCK) inhibitors promote corneal endothelial cell proliferation, migration, and adhesion while limiting apoptosis, and have shown benefit both as monotherapy in early-stage disease and as an adjunct to Descemet stripping procedures and cataract surgery, reducing postoperative endothelial cell loss in affected eyes [52]. The underlying clinical evidence base for this claim is still maturing: a systematic review identified 17 clinical investigations of ROCK inhibitors in corneal disease, of which only one was a randomized, double-masked trial (netarsudil versus placebo in 29 eyes with FECD), four were non-randomized prospective cohort studies, three were retrospective observational cohorts, and the remainder were small case series or single-patient case reports-a literature dominated by small, uncontrolled, or single-arm studies rather than the large randomized trials available for anti-VEGF or complement-targeted therapies discussed elsewhere in this review [53]. Reported outcomes (corneal thinning, endothelial cell density, visual acuity gains of two or more lines) have been consistent across these studies, but the overall risk of bias was rated moderate for the non-randomized studies, and larger controlled trials are needed before ROCK inhibitors can be considered established therapy for FECD rather than a promising adjunct.

### 5.2. Complement-Targeted and Precision Anti-Angiogenic Therapeutics

Parallel to the mitochondrial- and receptor-based strategies described above, a distinct and more clinically mature therapeutic frontier has emerged directly from the complement and angiogenic mechanisms discussed earlier in this review. In AMD, complement inhibition targeting C3 (pegcetacoplan) and C5 (avacincaptad pegol) represents the first mechanism-specific pharmacologic approach to geographic atrophy, a form of advanced dry AMD for which no effective treatment previously existed [11,12,13]. Regulatory status differs by region: both agents received FDA approval in the United States, but the European Medicines Agency refused marketing authorization for pegcetacoplan in 2024, concluding that the magnitude of benefit did not outweigh the potential risks, and avacincaptad pegol’s EU application was separately withdrawn by its sponsor the same year following unfavorable interactions with the EMA’s Committee for Medicinal Products for Human Use [39]; neither agent is currently approved in the European Union. Pegcetacoplan’s risks include a set of safety signals that emerged after FDA approval and were not observed in the pivotal trials: post-marketing reports to the American Society of Retina Specialists identified cases of intraocular inflammation and occlusive retinal vasculitis, some resulting in severe and, in some cases, irreversible vision loss, and the FDA label now carries a corresponding warning. The pivotal trials also showed a clear dose-dependent increase in exudative (neovascular) AMD conversion by month 24–12% with monthly dosing and 7% with every-other-month dosing, versus 3% with sham, requiring ongoing monitoring for a complication that itself threatens vision [12,54]. A treatment-focused review of this therapeutic class is incomplete without weighing these harms against the anatomical benefit described above. Because these agents slow anatomical lesion growth without fully restoring or protecting visual function in most treated eyes, current research has shifted toward combination regimens that pair complement inhibition with agents targeting the lipid-metabolism and immunosenescence pathways described above, on the reasoning that a multi-target approach is more likely to address the disease’s genuinely multifactorial pathogenesis than any single-mechanism therapy alone [40].

In DME, the therapeutic frontier is less about identifying new molecular targets—VEGF blockade remains highly effective—and more about extending the durability of an already-effective mechanism. Sustained-release drug-delivery platforms and single-administration gene therapies designed to provide continuous endogenous anti-VEGF expression are intended to reduce the treatment burden associated with chronic intravitreal dosing while preserving the anatomical and functional gains achieved with conventional anti-VEGF regimens [29]. As detailed in Section 3.3, these programs have not shown uniform outcomes to date: ADVM-022’s DME program was discontinued following a distinct safety signal, whereas 4D-150 and RGX-314 remain in active Phase 2 development for DME with, to date, more favorable reported safety profiles [29,30,31,32,33]. Should the latter approaches succeed in confirmatory trials, they would represent a meaningful advance not because they introduce a new mechanism of action, but because they address one of the most persistent practical barriers to sustained treatment benefit: the difficulty many patients face in adhering to frequent, indefinite injection schedules over years of chronic disease.

Across both conditions, AI-assisted treatment response prediction, described in the previous section, is increasingly being positioned as a complementary layer atop these mechanism-targeted therapies rather than a standalone diagnostic tool. The ability to forecast which patients are likely to respond well to a given complement inhibitor, anti-VEGF agent, or corticosteroid before treatment begins could meaningfully shorten the trial-and-error period that currently characterizes much of retinal disease management, particularly for patients with DME whose baseline characteristics are heterogeneous enough that no single first-line agent performs optimally for all patients [49]. This need is particularly acute in DME, where a systematic review and bivariate meta-analysis of AI models predicting anti-VEGF treatment response (six studies, 427 participants) reported a pooled sensitivity of 86.4% (95% CI: 82.1–90.1%), specificity of 77.6% (95% CI: 72.8–82.0%), and a summary AUC of 0.89 (95% CI: 0.84–0.93), with moderate heterogeneity across studies (I2 = 45.2% for sensitivity)-a genuinely strong result, though still short of implying routine-care readiness, and awaiting further prospective, real-world validation before broad clinical deployment [49].

All of the approaches described in this section-mitochondrial-targeted and Rho-kinase support for FECD, complement inhibition and durable anti-VEGF delivery for AMD and DME, and AI-assisted treatment response prediction across both-are united by a common principle: intervening in the pathological process earlier and at a more mechanistic level than conventional broad-spectrum therapy allows, within evidence generated specifically for the four conditions that are the focus of this review. Table 3 summarizes these four strategies alongside their molecular target, developmental stage, evidence level, and associated harms.

## 6. Discussion

Table 1 formalized the strength of evidence linking each of the four conditions to the four mechanistic domains examined in this review. Beyond the domain-by-domain grading captured there, the domains themselves are not fully independent: oxidative stress arising from mitochondrial damage, for instance, can promote local inflammation, while inflammation can in turn destabilize both mitochondrial function and barrier integrity.

This mechanistic interconnection is not limited to the anterior–posterior comparisons already discussed. The complement and lipid-metabolism pathways implicated in AMD, for example, plausibly interact with the same oxidative and mitochondrial stress pathways central to FECD and DME, since oxidative damage is known to both trigger and be amplified by complement activation in retinal pigment epithelial cells [9]. Similarly, the neuroinflammatory injury pattern that differentiates AQP4-ON from MOG-ON at the level of RNFL and ganglion cell loss shares immunological features with the chronic, unresolved inflammation described in AMD, even though the two conditions differ enormously in tempo-optic neuritis unfolds over days to weeks, while AMD develops over years to decades [16,40]. Recognizing these shared upstream vulnerabilities, rather than treating each condition as pathophysiologically isolated, is likely to be increasingly important as combination and multi-target therapies move from preclinical development into clinical practice.

Stated plainly, the evidentiary matrix in Table 1 makes two testable predictions rather than serving only as a descriptive summary. First, where a domain is graded Strong or Moderate for a given condition, a biomarker or intervention validated in that domain for one condition should show at least a preliminary signal when tested in another condition sharing that same domain-level grade (for example, mitochondrial-targeted interventions shown to help in FECD would be predicted to show some benefit in DME’s mitochondrial component, given both are graded at least Moderate in that domain). Second, the matrix predicts that conditions sharing no domain in common (such as FECD and antibody-mediated optic neuritis, per Table 1) should not respond to the same mechanism-specific therapy, since this review found no shared strong domain between them. The framework would be undermined—not merely refined—if cross-condition biomarker or treatment signals were found to track poorly with the domain-level grades in Table 1, or if a therapy targeting a domain graded Strong in one condition showed no signal whatsoever when tested in a second condition graded Strong in that same domain; either finding would suggest the domain categories themselves, rather than genuine shared biology, are driving the apparent convergence.

These convergent pathways also create practical opportunities for diagnosing and treating ocular conditions. Structural imaging advances such as OCT and OCTA now allow clinicians to track tissue-level changes non-invasively over time. However, imaging alone does not capture the full physiological context in which these changes occur. Biomarkers capable of detecting disease presence and progression add a complementary layer of information: the preliminary bioimpedance association discussed in Section 3.3, if replicated, would illustrate how purely ocular evaluation can miss a meaningful systemic contribution to local pathology, though this remains an open question rather than an established finding.

Artificial intelligence-assisted image analysis adds a further layer to this diagnostic picture, though its role should be understood as still maturing rather than fully established. Where OCT and OCTA provide the raw structural data, AI models offer the potential to interpret that data faster, more consistently, and potentially with greater sensitivity to subtle early change than manual grading allows. However, the same limitations that apply to any predictive model trained on retrospective data apply here: performance depends heavily on the diversity and quality of training data, and the field has not yet established consistent standards for prospective, real-world validation across the diverse patient populations and imaging platforms in clinical use [46,51,55]. For the DME anti-VEGF-response literature specifically, the same systematic review cited above, drawing on its full 18-study qualitative pool (a broader population than the 6-study/427-participant subset used for the pooled sensitivity/specificity/AUC estimates just described), found that 89% of these 18 studies were retrospective, only 53% had undergone any external validation, and a substantial minority showed methodological red flags associated with spectrum bias and overfitting—including both eyes per patient inclusion without statistical adjustment for clustering in 39% of studies, small test sets relative to model complexity in 33%, and at least one study reporting an implausibly high AUC (>0.99) suggestive of data leakage [49]. Reporting quality in this literature would benefit from consistent application of AI-specific frameworks such as the Checklist for Artificial Intelligence in Medical Imaging (CLAIM) and the TRIPOD+AI statement for clinical prediction models, alongside STARD-AI, an AI-specific extension of standard diagnostic-accuracy reporting guidance that remains under development at the time of writing [56,57]. Clinicians and researchers should therefore treat current AI diagnostic tools as a valuable adjunct to, rather than a substitute for, the mechanism-based structural and systemic biomarkers discussed throughout this review.

Taken together, the literature supports early intervention as the most effective strategy for limiting disease progression across these conditions. Conventional therapies for FECD, DME, and optic neuritis are typically applied only after substantial structural damage has occurred. Newer approaches-including support of mitochondrial function, receptor modulation, and complement inhibition, alongside adjunct strategies discussed in Appendix A-could in principle be applied earlier, while injury remains reversible. Realizing this shift, however, depends on developing more sensitive biomarkers capable of detecting subclinical disease activity. Given that the global burden of these conditions is already substantial and projected to grow considerably over the coming decades, the cumulative population-level benefit of shifting even a fraction of patients from late-stage to early-stage intervention is likely to be considerable [20].

This narrative review carries the limitations typical of the format. It does not follow the systematic search and quality appraisal procedures of a formal systematic review, and the search strategy described in Section 2, while more detailed and reproducible than in the original submission (Table S1), remains non-systematic in the formal sense; this creates a risk of selection bias, particularly toward literature indexed in English and toward publications from a limited set of journals. Consistent with this limitation, we note that the final 58-item reference list is weighted toward open-access publisher venues (approximately 31% of references are published in MDPI journals) and toward Elsevier-published venues (approximately 24%, including the Lancet family of journals), with the remainder distributed across Springer Nature, Frontiers, Wiley/Cochrane, and several other publishers; by study design, the list combines a minority of large randomized controlled trials (approximately 10%) with a majority of narrative/mechanistic reviews, observational studies, and preclinical reports, a composition we consider appropriate for a mechanism-focused narrative review but disclose here for transparency. Language restriction to English-language sources is a further limitation, as is the absence of formal quality appraisal (e.g., risk-of-bias scoring) of the individual studies cited. This review was also not conducted under a registered protocol, and both study screening and the domain-strength grading in Table 1 were performed by a single reviewer rather than duplicated and reconciled by two independent raters; a formal measure of inter-rater agreement is therefore not available, and the grading in Table 1 should be read with that in mind. As detailed in Section 3.1, the ‘Not identified in this review’ tier used in Table 1 should be read as a statement about this review’s non-systematic sample, not as a claim that no evidence exists in the broader literature for that domain-condition pairing.

Several of the newer therapeutic strategies discussed, particularly the adjunct approaches described in Appendix A, are supported only by preliminary or preclinical studies and require validation in controlled clinical trials. Similarly, while AI-assisted diagnostic tools show considerable promise, most of the supporting evidence to date derives from retrospective analyses of curated datasets rather than prospective clinical validation, and readers should interpret reported accuracy figures accordingly [46]. Readers should weigh the strength of evidence behind each mechanism individually, distinguishing those with strong mechanistic and clinical support from those still at an early experimental stage.

## 7. Conclusions

Treatment of complex ocular disorders benefits from moving away from one-size-fits-all approaches toward strategies that target specific disease mechanisms. Whether the goal is limiting mitochondrial degeneration in the cornea, preserving blood–retinal barrier integrity, or protecting neuroretinal structure under chronic inflammation and complement dysregulation, effective therapy depends on the use of objective structural biomarkers. Pairing diagnostic advances such as OCT/OCTA and AI-assisted image analysis with mechanism-targeted therapy is a promising direction for maintaining cellular homeostasis, protecting tissue structure, and reducing the global burden of preventable blindness; though, as discussed above, the AI evidence base remains predominantly retrospective and not yet prospectively validated, and even the most mechanistically targeted therapies reviewed here (the complement inhibitors) show a persistent gap between anatomical and functional benefit rather than a clean translation from mechanism to clinical outcome.

The scale of this challenge is considerable: with hundreds of millions of people worldwide currently affected by vision impairment and blindness, and that number projected to grow substantially in the coming decades, even incremental improvements in early detection and mechanism-targeted intervention are likely to translate into meaningful reductions in global disability [18,20].

Further progress will depend on prospective studies that combine structural imaging and systemic biomarkers with clinical trials of the treatment strategies discussed here. As understanding of mitochondrial, vascular, complement-mediated, and neuroinflammatory mechanisms continues to grow, and as AI-assisted diagnostic tools mature alongside gene- and complement-targeted therapeutics, the field has a genuine and increasingly tractable opportunity to shift from reactive to preemptive management of ocular disease.

## Figures and Tables

**Figure 1:**
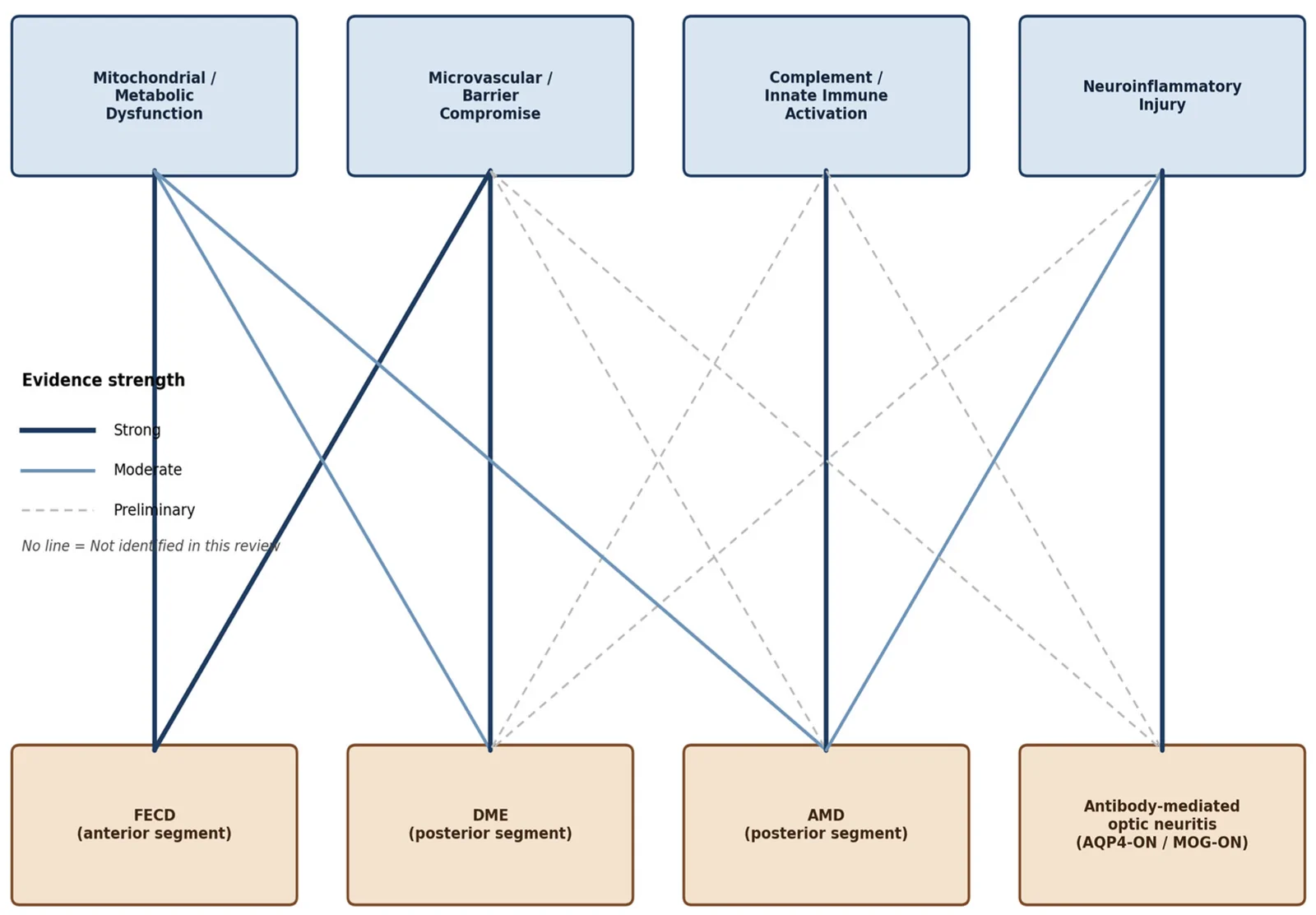
Schematic mapping of the four proposed convergent mechanistic domains onto the four ocular and neuro-ophthalmic conditions reviewed. Line weight and style indicate evidence strength (solid heavy = Strong, solid moderate = Moderate, dashed light = Preliminary); absence of a line indicates no substantive evidence was identified for that domain-condition pairing in this review. Corresponds to the assessment in Table 1.

**Table 1:** Evidentiary strength of four proposed convergent mechanistic domains across the four conditions reviewed. Bracketed numbers are representative citations; see main text for full support.

Condition	Mitochondrial/Metabolic Dysfunction	Microvascular/Barrier Compromise	Complement/Innate Immune Activation	Neuroinflammatory Injury
Fuchs endothelial corneal dystrophy (FECD)	Strong direct evidence in human corneal endothelium (mitochondrial stress, UPR activation) [1,2,3]	Strong-endothelial pump (Na+/K+-ATPase) failure and loss of deturgescence directly produce the corneal stromal edema that defines clinical FECD; the microvascular component of this domain does not apply, as the cornea is avascular [1]	Not identified in this review	Not identified in this review
Diabetic macular edema (DME)	Moderate-mitochondrial fission/mitophagy failure shown mechanistically upstream of endothelial injury generally, based on human-relevant mechanistic review evidence [4] and a rat retinal endothelial cell model [5]; a direct causal role specifically in tight-junction breakdown has not been established with equivalent evidence	Strong-VEGF-driven tight-junction failure, supported by pivotal human RCTs [6,7,8]	Preliminary-indirect, via broader diabetic microvascular inflammation, not disease-defining	Preliminary-limited direct human evidence specific to DME
Age-related macular degeneration (AMD)	Moderate-oxidative/mitochondrial stress implicated but less directly demonstrated than in FECD [9]	Preliminary-vascular contribution debated, secondary to complement/lipid pathways	Strong-genetic (CFH/CFI) and immunohistochemical human evidence; approved therapies exist [9,10,11,12,13]	Moderate-chronic microglial activation (‘inflammaging’), human and model evidence [9]
Antibody-mediated optic neuritis (AQP4-ON/MOG-ON)	Not identified in this review	Preliminary-reduced capillary vessel density shown in a mouse model during inflammatory episodes; not yet validated in human disease [14]	Preliminary-complement implicated in AQP4-ON astrocytopathy specifically, not MOG-ON	Strong-direct antibody-mediated axonal/ganglion cell injury, human clinical and OCT evidence [15,16,17]

Evidence tiers: Strong = direct evidence in the relevant human cell type or tissue, including clinical trial data where applicable; Moderate = mechanistic or indirect human evidence; Preliminary = limited, indirect, or largely preclinical evidence; Not identified in this review = no substantive evidence identified in the non-systematic literature search underlying this review (see Section 3.1). AQP4-ON = aquaporin-4 antibody-mediated optic neuritis; MOG-ON = myelin oligodendrocyte glycoprotein antibody-mediated optic neuritis; UPR = unfolded protein response.

## Data Availability

No new data were created in this review. All data discussed are available within the cited references. The supplementary search strategy is provided in Table S1 and the adjacent-therapy evidence summary in Table S2.

## Supplementary Materials

The following supporting information can be downloaded at: https://www.mdpi.com/article/10.3390/life16101624/s1, Table S1: Supplementary search strategy: topic clusters, representative Boolean search strings, and databases searched. Provided for reproducibility. Per-string yields, field-restriction tags, and publication-date limits applied are not separately logged, and this table does not distinguish which results derive from the initial November 2025 search versus the August 2026 re-run and extension described in the main text of Section 2; both runs used substantively the same strategy shown here. Approximate record counts by stage are reported in the main text of Section 2; Table S2. Adjacent anterior-segment and general ocular pharmacological strategies discussed in Appendix A, with disease applications outside the four index conditions of this review [41,42,58].

## Appendix A. Adjacent Anterior-Segment and General Ocular Pharmacology (Outside the Four Index Conditions)

The strategies discussed in this appendix act on pathways that recur across the four index conditions of this review (oxidative stress, innate immune activation, mitochondrial quality control) but have their primary supporting evidence in ocular conditions or tissues outside FECD, DME, AMD, and antibody-mediated optic neuritis specifically. They are presented here, separated from the main therapeutic discussion in Section 5, consistent with the principle that adjacent-pathway strategies without evidence in the index conditions should be clearly separated from the core evidence base rather than presented alongside it.

### Appendix A.1. Endogenous Modulators and Receptors

Although ocular surface disease and dry eye are not among the four conditions that are the primary focus of this review, endogenous opioid signaling and phytochemical anti-inflammatory strategies are included here because they act on the same anterior-segment tissue affected by FECD and target overlapping oxidative and inflammatory pathways discussed throughout this review; they are presented as adjacent, receptor- and pathway-based strategies relevant to anterior-segment ocular pharmacology more broadly, rather than as therapies specific to the four index conditions. A range of classical and non-classical opioid receptors is present on the ocular surface and associated nerves. These receptors help control local immune responses, cell proliferation, and pain. The corneal epithelium and nerve endings produce endogenous enkephalins that may have a role in treating chronic ocular surface disease, severe dry eye, and impaired wound healing when anti-inflammatory eye drops prove insufficient or carry excessive risk [58].

### Appendix A.2. Metabolic and Polyamine Treatment

Compounds that prolong cell viability and promote autophagy represent a preclinical avenue of interest for eye disease. For example, spermidine has shown protective effects against retinal ganglion cell loss in preclinical/animal models of glaucoma, optic nerve injury, and cataract by protecting these cells from oxidative stress [41]; no clinical efficacy has yet been demonstrated. We note explicitly that this evidence base is drawn from glaucoma models even though glaucoma itself was excluded from this review’s four index conditions (see Introduction); we consider this appropriate because the claim being made here concerns a mechanistic pathway (autophagy/mitochondrial quality control) rather than glaucoma as a disease, but readers should weigh this alongside the sponsoring organization’s disclosed glaucoma pipeline program (see Conflicts of Interest) in judging how much weight to give it. Given its proposed mechanism of action-promoting autophagic flux and improving mitochondrial quality-a similar benefit in the anterior segment (e.g., in FECD) is plausible, though this remains to be tested directly, including in human trials, and no such trial is presently underway to our knowledge.

### Appendix A.3. Phytochemistry and Ethnopharmacology

Botanical anti-inflammatory agents are included in this review for the same reason as the opioid-receptor material above: general ocular inflammation targets pathways (oxidative stress, innate immune activation) that recur across the four index conditions, even though the botanical evidence itself has not yet been tested specifically in FECD, DME, AMD, or antibody-mediated optic neuritis. The intersection of traditional botanical medicine with modern drug-delivery systems represents a large and still largely untapped source of anti-inflammatory biological agents. Rigorous preclinical studies of botanical extracts are needed to identify novel leads capable of inhibiting ocular inflammation without unacceptable toxicity [42]. Dose–response analysis and toxicological evaluation of such candidates will be necessary before any direct comparison with conventional pharmacologic agents.
