# Supplementary material for: Bridging Mechanism and Therapy in Ocular Disease: Mitochondrial, Vascular, and Immune Pathways in the Eye
Source: Life (Basel). 2026 Sep 28;16(10):1624. doi: 10.3390/life16101624 (PMC13705779; doi:10.3390/life16101624)
Supplement: Supplementary file 1 [file life-16-01624-s001.zip › life-4479103-supplementary.pdf]

Table S1. Supplementary search strategy: topic clusters, representative Boolean search strings, and databases searched. Provided for reproducibility in response to reviewer request.

Topic cluster	Representative Boolean search string (adapted per database syntax)	Databases searched
FECD / corneal endothelium	("Fuchs endothelial corneal dystrophy" OR "FECD") AND ("mitochondri*" OR "mitophagy" OR "unfolded protein response" OR "TCF4" OR "CTG18.1")	PubMed/MEDLINE; Embase; Cochrane Library
DME / blood-retinal barrier	("diabetic macular edema" OR "diabetic macular oedema") AND ("blood-retinal barrier" OR "tight junction" OR "VEGF" OR "mitophagy" OR "Fis1" OR "Drp1")	PubMed/MEDLINE; Embase; Cochrane Library
AMD / complement / ECM	("age-related macular degeneration" OR "AMD") AND ("complement" OR "CFH" OR "CFI" OR "geographic atrophy" OR "drusen" OR "extracellular matrix" OR "reelin" OR "inflammaging")	PubMed/MEDLINE; Embase; Cochrane Library
Optic neuritis / neuroinflammation	("optic neuritis" OR "AQP4" OR "MOG" OR "MOGAD" OR "neuromyelitis optica") AND ("OCT" OR "retinal nerve fiber layer" OR "ganglion cell")	PubMed/MEDLINE; Embase; Cochrane Library
Imaging / AI diagnostics	("optical coherence tomography" OR "OCT angiography" OR "OCTA") AND ("deep learning" OR "artificial intelligence" OR "machine learning" OR "convolutional neural network")	PubMed/MEDLINE; Embase
Complement / gene therapy	("pegcetacoplan" OR "avacincaptad" OR "complement inhibitor") OR ("gene therapy" AND ("AAV" OR "RGX-314" OR "ADVM-022" OR "4D-150"))	PubMed/MEDLINE; Embase; Cochrane Library
Epidemiology / global burden	("global burden of disease" OR "GBD") AND ("blindness" OR "vision impairment" OR "visual impairment")	PubMed/MEDLINE

Search strings are representative rather than exhaustive; syntax was adapted to each database's field-search conventions (e.g., MeSH mapping in PubMed, Emtree mapping in Embase). Approximate record counts by stage are reported in the main text of Section 2.

Table S2. Adjacent anterior-segment and general ocular pharmacological strategies discussed in Appendix A, with disease applications outside the four index conditions of this review.

Strategy	Primary Molecular Target	Disease Application	Developmental Stage	Evidence Level	Harms / Safety Considerations	Reference (s)
Endogenous opioid receptor modulation	mu/delta/kappa opioid receptors, ocular surface nerves	Chronic ocular surface disease, dry eye	Early clinical/preclinical	Preliminary (early clinical/preclinical)	Limited human safety data; systemic opioid-related risks not expected at topical ocular doses studied to date, but not formally characterized	[41]

Polyamine (spermidine) supplementation	Autophagy/mitochondrial quality control	Glaucoma, optic nerve injury, cataract (preclinical/animal models)	Preclinical	Preliminary (preclinical/animal only)	No ocular clinical safety data; oral spermidine has a favorable safety record in non-ocular supplement use, which does not establish ocular safety	[42]
Botanical/ethnopharmacological anti-inflammatories	Multiple (inflammation resolution)	General ocular inflammation	Preclinical	Preliminary (preclinical only)	Toxicological evaluation not yet established for most candidates; dose-response and safety data required before human testing	[58]

Evidence Level tiers as defined in Table 1. These strategies are not restricted to, and in most cases have not been tested within, the four index conditions of this review; they are included for pathway relevance only, consistent with the rationale given in Appendix A.
